# Supplementary material for: Translation and cross-cultural adaptation of the Persian version of inflammatory bowel disease-fatigue (IBD-F) self-assessment questionnaire
Source: PLoS One. 2023 Jul 21;18(7):e0288592. doi: 10.1371/journal.pone.0288592 (PMC10361485; doi:10.1371/journal.pone.0288592)
Supplement: S1 Table — (DOCX) [file pone.0288592.s001.docx]

| **Supplementary Table 1.** Past medical and drug history of the participants. | | | |
| --- | --- | --- | --- |
| Variables  (number (%) or  mean ± SD) | Study Groups | | |
|  | All Patients  (N=54) | CD patients  Group (N=26, 48%) | UC Patients  Group (N=28, 52%) |
| *Past medical history* |  |  |  |
| *Patients without comorbidities* | 36 (67%) | 17 (65%) | 19 (68%) |
| *Cardiovascular disorders* | 3 (6%) | 3 (12%) | - |
| *Respiratory disorders* | 2 (4%) | - | 2 (7%) |
| *Gastrointestinal disorders ** | 6 (11%) | 2 (8%) | 4 (14%) |
| *Thyroid disorders* | 5 (9%) | 2 (8%) | 3 (11%) |
| *Cerebrovascular disorders* | 2 (4%) | - | 2 (7%) |
| *Psychiatric disorders* | 4 (8%) | 1 (4%) | 3 (11%) |
| *Reproductive & Urinary system disorders* | 2 (4%) | 2 (8%) | - |
| *Iron deficiency anemia* | 37 (69%) | 18 (69%) | 19 (68%) |
| *Vitamin-D Deficiency* | 19 (35%) | 10 (39%) | 9 (32%) |
| *Covid-19* | 30 (56%) | 16 (62%) | 14 (50%) |
| *Past drug history* |  |  |  |
| *Without medication history *** | 7 (13%) | 2 (8%) | 5 (18%) |
| *5-aminosalicylic acid* | 26 (48%) | 9 (35%) | 17 (61%) |
| *Steroid* | 6 (11%) | 2 (8%) | 4 (14%) |
| *TNF inhibitor* | 27 (50%) | 16 (61%) | 11 (39%) |
| *Immunosuppressant* | 16 (30%) | 7 (27%) | 9 (32%) |
| [*Vitamin & Mineral Supplements*](https://www.bing.com/ck/a?!&&p=0d8c4a7dc94abf16JmltdHM9MTY2NzQzMzYwMCZpZ3VpZD0yMmIwYzRhMi04ODE1LTY3YzMtMTExNC1kNDAxODljNzY2OWUmaW5zaWQ9NTE5Nw&ptn=3&hsh=3&fclid=22b0c4a2-8815-67c3-1114-d40189c7669e&psq=minreal+and+vitamin+supplement&u=a1aHR0cHM6Ly93d3cubnV0cml0aW9uLmdvdi90b3BpY3MvZGlldGFyeS1zdXBwbGVtZW50cy92aXRhbWluLWFuZC1taW5lcmFsLXN1cHBsZW1lbnRz&ntb=1) | 19 (35%) | 9 (35%) | 10 (36%) |
| *Other* | 10 (19%) | 4 (15%) | 6 (21%) |
| CD, Crohn's disease; N, Number; UC, Ulcerative colitis.  * Any other gastrointestinal disorder except inflammatory bowel disease.  ** It displays patients who are not taking any pills. | | | |
