# Supplementary material for: Translation and cross-cultural adaptation of the Persian version of inflammatory bowel disease-fatigue (IBD-F) self-assessment questionnaire
Source: PLoS One. 2023 Jul 21;18(7):e0288592. doi: 10.1371/journal.pone.0288592 (PMC10361485; doi:10.1371/journal.pone.0288592)
Supplement: S1 File — This file contains the Persian version of the IBD-F questionnaire and can be used by native Persian researchers and physicians for the measurement of fatigue in IBD patients. (DOCX) [file pone.0288592.s002.docx]

مقیاس خود ارزیابی بیماری التهابی روده-خستگی (IBD-F)

تاریخ: ............. زمان: ................

بخش 1-مقیاس ارزیابی خستگی

این بخش از پرسشنامه خستگی، شدت، تکرر و مدت زمان آن را شناسایی می باشد.

افراد مبتلا به بیماری التهابی روده گاهی احساس خستگی می کنند. واژه «خستگی» در سراسر پرسشنامه به کار رفته و به صورت خستگی مداوم با دوره هایی ناگهانی و طاقت فرسا از احساس فقدان انرژی یا تخلیه شدن از انرژی تعریف می شود که با استراحت یا خواب بهبود پیدا نمی کند.

| لطفا برای هر سوال «یک» عدد را علامت بزنید | امتیازات از 0 تا 4  0= عدم احساس خستگی |  | | | 4=احساس خستگی شدید |
| --- | --- | --- | --- | --- | --- |
| 1. در حال حاضر چقدر احساس خستگی می کنید. | 0 | 1 | 2 | 3 | 4 |
| 1. بالاترین سطح احساس خستگی شما در دو هفته گذشته چقدر بوده است. | 0 | 1 | 2 | 3 | 4 |
| 1. پایین ترین سطح احساس خستگی شما در دو هفته گذشته چقدر بوده است. | 0 | 1 | 2 | 3 | 4 |
| 1. میانگین سطح احساس خستگی شما در دو هفته گذشته چقدر بوده است. | 0 | 1 | 2 | 3 | 4 |
| 1. در دو هفته گذشته چه میزان از زمان هایی که از خواب بیدار شدید احساس خستگی داشتید. | 0  هیچ وقت | 1  بعضی اوقات | 2  خیلی اوقات | 3  بیشتر اوقات | 4  همیشه |

بخش 2- مقیاس اثر بیماری التهابی روده-خستگی روی فعالیت های روزانه

این بخش درک شما از میزان تأثیر خستگی بر روی فعالیت های روزانه ی خود را در طول دو هفته گذشته مورد ارزیابی قرار می دهد.

لطفا به کلیه سوالات پاسخ دهید. پاسخ های احتمالی به سوالات عبارتند از: هیچ وقت-0، بعضی اوقات-1، خیلی اوقات-2، بیشتر اوقات-3، همیشه-4.

در صورتی که یک فعالیت خاص درمورد شما صدق نمی کند (برای مثال رانندگی نمی کنید) گزینه N/A (not applicable) را انتخاب نمایید.

| برای هر سوال لطفا فقط یک گزینه که نشان دهنده وضعیت دو هفته گذشته شما باشد را انتخاب نمایید. | هیچ وقت | بعضی اوقات | اغلب اوقات | بیشتر اوقات | همیشه | صدق نمی کند (N/A) |
| --- | --- | --- | --- | --- | --- | --- |
| 1. بدلیل احساس خستگی باید در طول روز چرت می زدم | 0 | 1 | 2 | 3 | 4 |  |
| 1. خستگی مانع بیرون رفتن من و حضور در رویدادهای اجتماعی شد. | 0 | 1 | 2 | 3 | 4 |  |
| 1. من بدلیل خستگی قادر به حضور در محل کار یا مدرسه نبودم. | 0 | 1 | 2 | 3 | 4 | صدق نمی کند |
| 1. عملکرد من در محل کار یا تحصیل تحت تأثیر خستگی قرار گرفته بود. | 0 | 1 | 2 | 3 | 4 | صدق نمی کند |
| 1. من بدلیل خستگی برای تمرکز کردن مشکل داشتم. | 0 | 1 | 2 | 3 | 4 |  |
| 1. بدلیل خستگی ایجاد انگیزه برایم سخت بود. | 0 | 1 | 2 | 3 | 4 |  |
| 1. بدلیل خستگی نمی توانستم به نظافت و آراستگی خود برسم. | 0 | 1 | 2 | 3 | 4 |  |
| 1. بدلیل خستگی راه رفتن برایم دشوار بود. | 0 | 1 | 2 | 3 | 4 |  |
| 1. بدلیل خستگی قادر به رانندگی به میزانی که لازم بود، نبودم. | 0 | 1 | 2 | 3 | 4 | صدق نمی کند |
| 1. بدلیل خستگی نتوانستم آنطور که میخواستم فعالیت ورزشی انجام دهم. | 0 | 1 | 2 | 3 | 4 |  |
| 1. بدلیل خستگی برای ادامه ی تفریحات/علایق خود مشکل داشتم. | 0 | 1 | 2 | 3 | 4 |  |
| 1. رابطه ی عاطفی من با شریک زندگیم بدلیل خستگی تحت تأثیر قرار گرفت. | 0 | 1 | 2 | 3 | 4 | صدق نمی کند |
| 1. رابطه ی جنسی من با شریک زندگیم بدلیل خستگی تحت تأثیر قرار گرفت. | 0 | 1 | 2 | 3 | 4 | صدق نمی کند |
| 1. رابطه ی من با فرزندانم بدلیل خستگی تحت تأثیر قرار گرفت. | 0 | 1 | 2 | 3 | 4 | صدق نمی کند |
| 1. بدلیل خستگی خلق من پایین بود. | 0 | 1 | 2 | 3 | 4 |  |
| 1. بدلیل خستگی احساس انزوا داشتم. | 0 | 1 | 2 | 3 | 4 |  |
| 1. بدلیل خستگی حافظه ی من تحت تأثیر قرار گرفت. | 0 | 1 | 2 | 3 | 4 |  |
| 1. بدلیل خستگی مرتکب اشتباه شدم. | 0 | 1 | 2 | 3 | 4 |  |
| 1. خستگی مرا تحریک پذیر نمود. | 0 | 1 | 2 | 3 | 4 |  |
| 1. خستگی باعث شد احساس ناامیدی کنم. | 0 | 1 | 2 | 3 | 4 |  |
| 1. بدلیل احساس خستگی کلمات را گم کردم. | 0 | 1 | 2 | 3 | 4 |  |
| 1. خستگی مانع احساس لذت از زندگی در من شد. | 0 | 1 | 2 | 3 | 4 |  |
| 1. خستگی مانع داشتن احساس رضایت از زندگی در من شد. | 0 | 1 | 2 | 3 | 4 |  |
| 1. عزت نفس من تحت تأثیر خستگی قرار گرفت. | 0 | 1 | 2 | 3 | 4 |  |
| 1. خستگی اعتماد به نفس من را تحت تأثیر قرار داد. | 0 | 1 | 2 | 3 | 4 |  |
| 1. خستگی باعث شد احساس خوشحالی نداشته باشم. | 0 | 1 | 2 | 3 | 4 |  |
| 1. بدلیل احساس خستگی شب ها برای خوابیدن با مشکل مواجه بودم. | 0 | 1 | 2 | 3 | 4 |  |
| 1. خستگی توانایی من برای انجام کلیه فعالیت های طبیعی من در منزل را تحت تأثیر قرار داد. | 0 | 1 | 2 | 3 | 4 |  |
| 1. بدلیل خستگی باید از دیگران درخواست کمک می کردم. | 0 | 1 | 2 | 3 | 4 |  |
| 1. کیفیت زندگی من تحت تأثیر خستگی قرار گرفت. | 0 | 1 | 2 | 3 | 4 |  |

بخش 3- سوالات تکمیلی درمورد احساس «خستگی» شما

1. بنظر شما غیر از بیماری التهابی روده دلیل اصلی احساس خستگی شما چیست؟.........................................................................

............................................................................................................................................................................................................................

............................................................................................................................................................................................................................

1. فکر می کنید سایر عللی که موجب احساس خستگی شما می شوند چیست؟................................................................................

............................................................................................................................................................................................................................

............................................................................................................................................................................................................................

1. آیا چیزی که به احساس خستگی شما کمک کند یافته اید؟...............................................................................................................

............................................................................................................................................................................................................................

............................................................................................................................................................................................................................

1. برای چه مدت زمانی احساس خستگی را تجربه کرده اید؟ ..............سال............ماه
2. در این دوره زمانی احساس خستگی شما به چه صورت بوده است: الف) مداوم ب)دوره ای
