# Supplementary material for: Translation and cross-cultural adaptation of the Persian version of inflammatory bowel disease-fatigue (IBD-F) self-assessment questionnaire
Source: PLoS One. 2023 Jul 21;18(7):e0288592. doi: 10.1371/journal.pone.0288592 (PMC10361485; doi:10.1371/journal.pone.0288592)
Supplement: Attachment — Submitted filename: Response to reviewers.docx [file pone.0288592.s003.docx]

Dear Editor and reviewers,

Hope everything goes well.

Great appreciation for your time and consideration. We have applied all your recommendations to make the manuscript better and more precise. Changes have been specified with highlights. I hope the responses to the obscure parts be helpful.

**Response to Journal Requirements:**

1. The manuscript style was changed based on PLOS ONE style requirements.
2. The manuscript was edited for language usage, spelling, and grammar by the institutional language editing service; A copy of the manuscript showing changes (with track changes and highlights) was uploaded as the supporting information file.
3. There were no legal or ethical restrictions on sharing data publicly; therefore, we uploaded our data set into the Figshare repository, and they are available online via **DOI: 10.6084/m9.figshare.23292446**. Furthermore, we revised our data availability statement at the end of the manuscript (line: 333-334).
4. The ethics statement was transferred from the declaration section into methods (lines 116-118).
5. Caption for supplementary files was added at the end of manuscript (lines 497-501).

**Response to Editor:**

1. Comment: I would like you to indicate how many doctors and researchers would utilize it when translated into Persian, and how much it would contribute to the evaluation of patients. Answer: The number of clinicians and researchers dealing with IBD and Fatigue who benefit from this cross-cultural adaptation is explained through lines 328-339. Besides, the innovation and importance of this project get more prominent when there used to be no valid and reliable questionnaire to measure fatigue in IBD patients in Iranian society. Also, the application of this questionnaire for screening, prioritizing, designing an intervention, and measuring the efficacy of the designed intervention is explained within lines 340-351
2. Comment: Furthermore, many fatigue-related scores have emerged, and in many clinical studies, FACIT-F has started to be used for patients with Inflammatory Bowel Disease (IBD). I would like you to further clarify why you chose FACIT-F for IBD. Answer: As you said, FACIT-F has been used in many clinical studies for patients with Inflammatory Bowel Disease (IBD); however, the reasons that IBD-F may seem to be more fruitful rather than FACIT-F for measuring fatigue are explained through lines 103-110 (designed explicitly for IBD patients, being more comprehensive, having more extended recall period, and owning a counseling section).
3. Comment: Please revise this sentence: However, none of them have been validated in the IBD population and are not specific to the problems of patients with IBD. Answer: Thanks for your valuable comment. FACIT and Fatigue questionnaires are validated for IBD patients; however, they comprise 13 and 20 items, respectively, to measure the severity and impact of fatigue on daily activities. IBD-F is a 35-item scale for measuring fatigue, so it can more comprehensively evaluate the severity and effects of fatigue. Thus, the previous sentence (lines 105 - 107) was revised to " Some of them have been validated in the IBD population; however, there was a need for a more comprehensive questionnaire that examined more precisely the effect of fatigue on the daily activity of IBD patients. "
4. Comment: Is this sentence true (Following the Bartlett recommendation (23), which declared that the minimum sample size for method comparison investigation is 50 subjects)? I cannot find this description in the cited article. Answer: As we figured out that mentioned sentence caused the misunderstanding, it was omitted, and the calculation of the sample size via G power version 3.1.9 was replaced (alpha-error: 0.05, beta-error: 0.05, power: 0.95, and effect size: 0.83 (based on correlation of the original IBD-F questionnaire)) (lines 184-187).
5. Comment: Careless mistakes are noticeable, such as forgetting to put spaces. Please correct them. Answer: The manuscript underwent the language editing services by institutional language editing service and all the efforts were made to correct the writing errors.

**Response to reviewer 1**:

1. Comment: Change this sentence in Abstract: Background: This is the first study to appraise the validity and reliability of the Persian version of the Inflammatory bowel disease-fatigue (IBD-F) questionnaire. Answer: Your mentioned sentence in the abstract (lines 30-31) was changed to " This study appraises the psychometrics properties of the Persian version of the Inflammatory bowel disease-fatigue (IBD-F) questionnaire."
2. Comment: Mention the innovation and importance of this research. Answer: The number of clinicians and researchers dealing with IBD and Fatigue who benefit from this cross-cultural adaptation is explained through lines 328-339. Besides, the innovation and importance of this project get more prominent when there used to be no valid and reliable questionnaire to measure fatigue in IBD patients in Iranian society.
3. Comment: Add related references for lines 122-123, 131-132. Answer: A reference for lines (120-121, previous line number 122-123) was added that illustrates how the standard forward and backward was carried out (doi: 10.1111/j.1524-4733.2005.04054.x. PubMed PMID: 15804318); furthermore, for lines (128-129, previous line numbers 131-132) we demonstrated that mean of " compared with the original issue " was the first version of IBD-F questionnaire (doi: 10.1016/j.crohns.2014.04.013. PubMed PMID: 24856864).
4. Comment: In the method section add the Population and statistical sample. Answer: Calculation of sample size via G* Power software from the IBD patients’ population was explained within lines 184-185.
5. Comment: Report the results of confirmatory factor analysis. Answer: Other transcultural versions of the IBD-F questionnaire (like Polish doi: 10.5114/pg.2021.106665, and Danish PubMed PMID: 28869029) did not undergo the CFA because they evaluated the validity and reliability without any dimension reduction (omitting some items). We acted exactly like them; however, based on your recommendation, CFA was done, via LISREL version 8, to evaluate the construct validity within lines 177-181 and 236-238. The results show the relative property (borderline results) of observed data to the model fitness; however, the low ratio of sample size (54 patients) to the number of items (35 questions) may affect the construct validity analysis, and with a more comprehensive sample size a finer result of fitness will be achieved; thus, this issue cannot be directly related to the questionnaire, and it was because of limitation in methodology (sample size).
6. Comment: The limitations and applications of the study should be mentioned. Answer: The application of this questionnaire for screening, prioritizing, designing an intervention, and measuring the efficacy of the designed intervention is explained within lines 340-351. Also, the current study's limitations were mentioned in lines 352-361.

**Response to reviewer 2:**

Comment: The manuscript is ok in the current form for publication. Although, the authors were used the classical methods of psychometric but they could use Rasch analysis for approving psychometric properties and it would be more robust than the previous one. Answer: Thanks for your precious comment. Classical psychometrics and Rasch analysis are two different approaches used in the transcultural adaptation of a questionnaire. Classical methods are used to explore the underlying factor structure of a questionnaire. In contrast, Rasch analysis is used to model the relationship between a person's responses to the questionnaire items and the latent trait being measured. Rasch analysis is a newer method for evaluating transcultural adaption; however, both approaches have their strengths and weaknesses, and the choice of which method to use depends on the research question and the available data. We recommended that in a subsequent analysis article, the Rasch analysis can be performed to evaluate the transcultural adaption of the questionnaire (lines 355-357).

Makan Cheraghpour, Ph.D.

Shabnam Shahrokh MD.
